# Microbleeds in Late-Life Depression: Comparison of Early- and Late-Onset Depression

**DOI:** 10.1155/2014/682092

**Published:** 2014-02-26

**Authors:** Chao Feng, Min Fang, Yu Xu, Ting Hua, Xue-Yuan Liu

**Affiliations:** ^1^Department of Neurology, Shanghai Tenth People's Hospital of Tongji University, Middle Yanchang Road No. 301, Zhabei District, Shanghai 200072, China; ^2^Yiwu Affiliated Hospital of Zhejiang University School of Medicine, Zhejiang, China; ^3^Department of Radiology, Shanghai Tenth People's Hospital of Tongji University, Shanghai, China

## Abstract

Late-life depression could be classified roughly as early-onset depression (EOD) and late-onset depression (LOD). LOD was proved to be associated with cerebral lesions including white matter hyperintensities (WMH) and silent brain infarctions (SBI), differently from EOD. However, it is unclear whether similar association is present between LOD and microbleeds which are also silent lesions. In this study, 195 patients of late-life depression were evaluated and divided into EOD, presenile-onset depression (POD), and LOD groups; 85 healthy elderly controls were enrolled as controls. Subjects were scanned by MRI including susceptibility weighted images to evaluate white matter hyperintensities (WMH), silent brain infarctions (SBI), and microbleeds. The severity of depression was evaluated with 15-item Geriatric Depression Scale. Psychosocial factors were investigated with Scale of Life Events and Lubben Social Network Scale. Logistic regression and linear regression were performed to identify the independent risk factors for depression. Results showed that LOD patients had higher prevalence of microbleeds than EOD, POD, and control patients. The prevalence of lobar microbleeds and microbleeds in the left hemisphere was the independent risk factor for the occurrence of LOD; a high number of microbleeds were associated with severe state of LOD, whereas life events and lack of social support were more important for EOD and POD. All these results indicated that Microbleeds especially lobar microbleeds and microbleeds in the left hemisphere were associated with LOD but not with EOD.

## 1. Introduction

Depression is a common mood disorder among the elderly. According to the onset age of depression, depression of the elderly, that is, late-life depression (LID), could be classified as early-onset depression (EOD) and late-onset depression (LOD), with the cut-off age of onset being unclear and varying between 40 to 65 years [1, 2]. The etiologies of EOD and LOD might be different to some extent. Compared with EOD, LOD was proved to be associated with cerebrovascular diseases more strongly [3]. Several studies demonstrated that, compared with EOD, patients with LOD had higher prevalence of white matter hyperintensities (WMH) [4] and silent brain infarctions (SBI) [5] which are both silent lesions of cerebrovascular diseases. Besides, the occurrence and degree of WMH might also affect the symptom severity of depression [6]. Based on the above mentioned finding, some researchers proposed the “vascular depression” hypothesis as the etiological explanation for LOD [7, 8] and attributed LOD to silent lesions which could destruct neurons and connective fibers critical for mood control.

Microbleeds represent another subtype of silent lesions which indicate covert vascular damages, similar to WMH and SBI [9]. Recently, several studies showed that microbleeds were related to a higher rate and severity of poststroke depression (PSD) measured by Geriatric Depression Scale (GDS) [10, 11], similar to the association between WMH and LOD. However, the association between microbleeds and depression was only demonstrated among patients of PSD. It was reported that the occurrence of microbleeds in acute stroke was related to neurological deterioration [12] which might lead to unfavorable outcome and a high rate of subsequent depression; therefore, the association between microbleed and PSD might be mediated by stroke itself. No study examined the association between microbleeds and depression of generally elderly population. In the present study, we aimed to fill this gap and explore the possible relation between microbleeds and different subtypes of LID in the absence of stroke.

## 2. Methods

### 2.1. General Information

This was a cross-sectional study, approved by *the Ethics Committee of Shanghai Tenth People's Hospital. *All procedures were carried out after the consent of each subject and the family members.

From September 2011 to July 2012, patients who attended to the Department of Neurology and Psychiatry of 10th People's Hospital in Shanghai were screened and enrolled into this study according to the following criteria: (1) 65 years of age or older; (2) diagnosis of new onset or recurrent major depression according to the diagnostic and statistical manual of mental disorders 4th edition (DSM-IV); (3) minimental state examination (MMSE) score higher than 17, with ability and will to give consent to this study. Patients with a history of acute stroke, brain tumor, or other central nervous system disease, patients with severe somatic disease including cancer, uremia, respiratory failure, heart failure with a New York Heart Association degree of 3 or 4, cirrhosis, and lupus, and patients with drug dependence were excluded. All patients and their family members were questioned about the detail of previous episodes of depression, with their accessible medical records as complementary data. According to the time of first depressive episode, patients were divided into EOD group when they developed major depression before 50 years, presenile onset depression (POD) group when they had their first depressive episode between 50 to 65 years, and LOD group when they had their first depressive episode after 65 years. At the same time, a group of healthy subjects attending our hospital for routine health examination with age of 65 years or older, MMSE score higher than 17, without depression and the above mentioned diseases were enrolled as control. All subjects were enrolled consecutively according to the respective criteria.

### 2.2. Neuropsychosocial Assessment

All subjects were evaluated with a series of neuropsychosocial scales including MMSE, 15-item Geriatric Depression Scale (GDS), Scale of Life Events (SLE), and Lubben Social Network Scale (LSNS) by two examiners (MMSE and GDS by Ming Fang, SLE and LSNS by Chao Feng) during one visit. Considering that most subjects had education less than 6 years, the cut-off of MMSE score was set at a relatively low level to exclude moderate and severe cognitive dysfunction. GDS is widely used for the evaluation of depression and has been proved to be well validated among Chinese elderly [13]; a higher score of GDS suggests a severer state of depression. SLE is a detailed self-report scale consisting of 61 items of different life events which happened during the last year, with the catastrophic and upsetting ones on the top of the scale [14]; in this study, the total number of life events was recorded as the score of SLE. LSNS is designed for the evaluation of interactions between the elderly and their social network, consisting of 10 items of different aspects of social network, with the maximum score of 50 [15]; a lower score of LSNS indicates lack of social support. All the scales were performed by one research assistant.

### 2.3. Radiological Examination

All subjects were imaged by a 3.0T MR scanner (Siemens 3.0T Magnetom Verio, Germany). The MRI imaging protocol consisted of T1-weighted images (repetition time (*T*_R_)/echo time (*T*_E_) = 2000/9), FLAIR (*T*_R_/*T*_E_ = 8500/94), and diffusion-weighted imaging (*T*_R_/*T*_E_ = 6000/94) on the axial plane; T2-weighted images (*T*_R_/*T*_E_ = 4540/96) on the sagittal plane with a thickness of 5.5 mm; and susceptibility weighted images (SWI) (*T*_R_/*T*_E_ = 27/20) on the axial plane with a thickness of 1.5 mm.

The silent cerebral lesions were assessed by two radiologists (Yu Xu and Ting Hua) blind to the clinical information, respectively. The discrepancy was resolved by further discussion between them after which they would give a unanimous conclusion.

Microbleeds were defined as homogenous round areas of signal loss with diameters less than 10 mm on SWI images [16]. Hypointense lesions within the subarachnoid space, basal ganglia mineralization, and other lesions or structures with similar signals were excluded during the microbleeds evaluation. The presence of microbleeds in any region and the presence of lobar, deep, and infratentorial microbleeds were recorded, respectively. The numbers of microbleeds were further graded as follows: 0 for no microbleeds, 1 for 1 microbleed, 2 for 2–4 microbleeds, and 3 for more than 4 microbleeds [17].

WMH was defined as focal or confluent hyperintensities in the deep or periventricular area on FLAIR images [18]. The severity of periventricular and deep WMH was respectively scaled as from 0 to 3 according to Fazekas' scale [19]. SBI was defined as a focal cavitated lesion 3 mm to 15 mm in size, with hypointensity on T1-weighted images and hyperintensity on T2-weighted images, without corresponding stroke history [20].

### 2.4. Data Analysis

All data were analyzed with SPSS 18.0. Chi-square test and Student's *t*-test were respectively used to compare the values of all the baseline characteristics, image features, and results of all scales. Kappa test was used to evaluate the agreement between the conclusions of two radiologists. Binary logistic regressions and multivariate linear regression were used to identify the independent risk factors for the occurrence of depression and severe state of depression, respectively. Details of regression models were introduced in Results Section.  *P* < 0.05 was considered to indicate statistical difference.

## 3. Results

**Figure 1:**
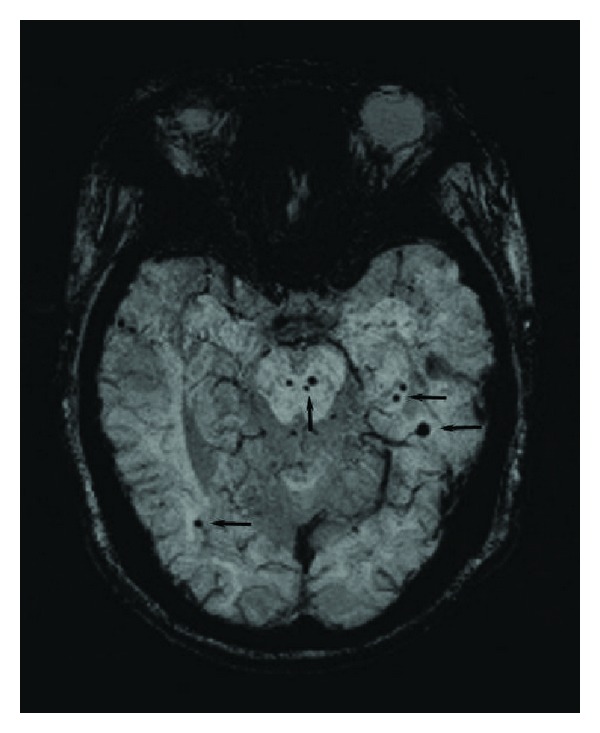
Examples of microbleeds detected by SWI. The black arrows pointed to lobar and infratentorial microbleeds.

57 patients with EOD, 54 patients with POD, 84 patients with LOD, and 85 healthy controls were enrolled. The baseline characteristics, image features, and results of all scales were listed in Table 1. Compared with control group, EOD and POD groups had lower LSNS and higher SLE scores, whereas LOD group had severer WMH, higher prevalence of SBI, and microbleeds, especially lobar microbleeds and microbleeds in the left hemisphere (Table 1). The comparisons between three depression groups showed that LOD group had lower MMSE and SLE, higher LSNS scores, higher WMH grade, higher frequency of SBI, and microbleeds, especially lobar microbleeds and microbleeds in the left hemisphere than EOD group, whereas the corresponding values of POD group were in a mediate position (details not shown). On the other hand, although the LOD group had the lowest MMSE scores, 19 patients still had normal MMSE scores (≥27), with lower prevalence of microbleeds especially lobar microbleeds and microbleeds in the left hemisphere, lower grade of PWMHm and also lower LSNS scores than others (*n* = 65) (all *P* < 0.05) (details not shown). The examples of microbleeds were shown in Figure 1.

The interrater reliability between two radiologists was determined by Kappa tests, whose results were 0.847 for the scores of PWMH, 0.913 for the scores of DWMH, 0.919 for the presence of SBI, and 0.915 for the presence of microbleeds. All of them suggested a high consensus.

Furthermore, logistic regression models were constructed to identify the independent risk factors of EOD, POD, and LOD, respectively, with the control group as contrast. The variables with *P* < 0.10 after comparison with control group as listed in Table 1 (except GDS score) were entered into the regression models. In detail, these models included one model for EOD, one model for POD, and three models for LOD with the prevalence of microbleeds in any region (*P* = 0.012 by Chi-square test as listed in Table 1), microbleeds in the left hemisphere (*P* = 0.001 in Table 1), and lobar microbleeds (*P* = 0.001 in Table 1) respectively analyzed. The results showed that a lower LSNS and a higher SLE were independent risk factors for both EOD and POD (details not shown), while a higher grade of DWMH, higher prevalence of microbleeds in the left hemisphere, and lobar microbleeds were independent risk factors for LOD. The results of logistic regressions for the occurrence of LOD were listed in Table 2.

The impact of microbleeds on the severity of depression, that is, GDS score, was analyzed with Student's *t*-test. The results showed that, in LOD group, patients with microbleeds in any region had significantly higher GDS scores than those without microbleeds (9.74 versus 7.82, *P* = 0.001). Besides, among those patients with microbleeds, patients with lobar microbleeds had even higher GDS scores than those without lobar microbleeds (10.19 versus 8.25, *P* = 0.007); patients with microbleeds in the left hemisphere also had higher GDS scores than those without microbleeds in the left hemisphere (10.11 versus 8.29, *P* = 0.018). However, in EOD and POD groups, no statistical difference of GDS scores was observed between patients with and without microbleeds (*P* > 0.05).

Furthermore, a multivariate linear regression model was constructed to identify the independent risk factors for a higher GDS score, that is, severe depression of LOD group, with age, sex, education, prevalence of hypertension, diabetes, SBI, degrees of PWMH and DWMH, MMSE, LSNS, and SLE scores, and the grade of microbleeds added into the model. The results showed that a higher degree of DWMH and a higher grade of microbleeds were independent risk factors for a higher GDS score. Details about the linear regression were listed in Table 3. Similar regression models were constructed in EOD and POD groups; however, none of the silent lesions were identified as the independent risk factors for a higher GDS score (details not shown).

## 4. Discussion

This study showed that microbleeds were associated with the occurrence and severity of LOD; microbleeds in the left hemisphere and lobes were more critical for LOD. However, similar association was not observed between microbleeds and EOD among the elderly.

This study supplied more proof in favor of the vascular hypothesis in the pathogenesis of LOD, which was mainly based on the previous studies of WMH and SBI, especially the former one [7]. Our study verified the roles of WMH and SBI again and found that DWMH might be more important than PWMH and SBI for LOD. Besides, the results suggested that microbleeds which represent covert vascular damages similar to WMH and SBI might also play important role in the pathogenesis of LOD in the absence of stroke and thus could be another pathological basis of the vascular hypothesis. The further analysis of lesion locations showed that lobar microbleeds and microbleeds in the left hemisphere were more critical than lesions in other regions for LOD. In fact, studies about PSD had similar findings, that is, patients with infarcts in some specific locations such as the left frontal and temporal lobes were more likely to develop PSD [21, 22]. Although it is still controversial, the lesion location hypothesis is now the most widely accepted etiological explanation for PSD. Some researchers concluded the critical regions for PSD as “frontal subcortical circuits” [23] or “limbic-cortical-striatal-pallidal-thalamic circuits” [24], which involved the neurons and fiber projections important for the emotional modulation. Our results about the lesion locations critical for LOD were also in accordance with the above mentioned circuits and emphasized the role of lesions in the left hemisphere and lobes again. This study also showed that DWMH rather than PWMH might be the independent risk factor for the occurrence and severity of LOD. It could also be explained by the lesion location hypothesis because DWMH involves the frontal subcortical circuits mentioned above.

Lobar microbleeds seemed to have stronger association with LOD. Interestingly, a recent study proved that lobar microbleeds also had stronger influence on cognitive dysfunction compared with microbleeds in other regions [17]. The strategic role of lobes in cognition and emotion might be a possible reason for their specialty. Besides, lobar microbleeds were proved to be related to cerebral amyloid angiopathy [25], different from WMH, SBI, and deep microbleeds which might be caused by hypertensive arteriolosclerosis [26]. Further studies are still needed to identify whether this pathological specialty contributes to the outstanding role of lobar microbleeds in LOD.

Compared with LOD, EOD seems to have different risk factors. We could not get the corresponding information including life events and cerebral lesions at the first depressive episode for them; however, the results still showed that accumulation of life events and lack of social support (lower LSNS scores) might be the main reasons for the recurrence of depression (attending to hospital) for patients with EOD, whereas the frequencies of three types of silent lesions (WMH, SBI, and microbleeds) were similar to those of control group; although compared with LOD group, they had longer duration of depression. It suggested that psychosocial factors including life events and social solitude were more important to EOD than silent cerebral lesions. Considering the different perspectives on the cut-off onset age of EOD and LOD, POD group was set as the transition from EOD to LOD, and the results about the risk factors of POD were also mediate between those of EOD and LOD. It suggested that the pathogenesis of POD might be more various, transiting from psychosocial factors to cerebral lesions.

In this study, we excluded patients with obvious cognitive impairment, whose presence could make the diagnosis of depression more difficult because it is sometimes similar to depression especially apathetic symptoms and might also make the conversation more difficult. However, without detailed investigation about the clinical characteristics, morphometric, or functional evaluation, patients in the early stage of vascular dementia, Alzheimer's disease, or other subtypes of cognitive impairment could not be totally excluded. Actually, cognitive impairment and depression could sometimes coexist especially in the elderly [27, 28] partly based on the same pathological mechanisms, such as the disruption of key regions by white matter and subcortical lesions [29]. This study showed that patients of LOD group had the most serious load of silent lesions including microbleeds; meanwhile, they had the lowest MMSE scores. It verified the association between depression and cognitive dysfunction again and suggested that microbleeds might also be the common etiological factor for both cognitive impairment and depression, similar to WMH.

On the other hand, part of patients still had high MMSE scores even in LOD group. The comparison within LOD group showed that patients with high MMSE scores had fewer microbleeds, lower grade of WMH, and lower LSNS scores. It verified the previous deduction that the cognitive dysfunction of LOD groups might be related to the silent lesions including microbleeds; meanwhile, it also suggested that, even for LOD, psychosocial factors were still important risk factors.

As a cross-sectional and single-centered study, its main strength involved the multiple groups including healthy controls which enabled the identification of risk factors for different types of depression and the utilization of 3.0T MRI and SWI which is better validated for the identification of microbleeds than traditional T2-GRE series [30]. However, some limitations were inevitable. First, the nonprospective study design limited the deduction of causality: we could only find the association between the factors evaluated in this study and depression and could not make a conclusion that silent lesions or life events resulted in depression. Second, mild cognitive dysfunction which could not be totally excluded might interfere with the diagnosis of depression. Third, the small sample size limited the significance of our results; meanwhile, the unequal sample sizes for different groups could only supply the proportions of different depression subtypes in one hospital but not the corresponding prevalence in community. Fourth, the grouping method based on the onset age of depression which could only be recalled and was not precise enough might also lead to some bias. However, we did find a close association between microbleeds and LOD in this study, whose results suggested that silent cerebral lesions especially DWMH, lobar microbleeds, and microbleeds in the left hemisphere were main determinants of the prevalence and severity of LOD, while psychosocial factors were more critical for EOD. Anyway, in the future, more studies are still needed to explore the pathogenesis of different types of depression.

## Figures and Tables

**Table 1:** Demographic, neuropsychosocial, and radiological characteristics of all subjects.

	Control (*n* = 85)	EOD (*n* = 57)	*P* (versus control)	POD (*n* = 54)	*P* (versus control)	LOD (*n* = 84)	*P* (versus control)
Age, years	72.25 ± 5.87	71.84 ± 4.95	0.669	73.06 ± 5.61	0.422	73.23 ± 4.78	0.236
Female	41 (48.2%)	37 (64.9%)	0.050	35 (64.8%)	0.056	53 (63.1%)	0.052
Education, years	4.25 ± 3.65	4.21 ± 4.18	0.956	3.56 ± 2.82	0.238	3.99 ± 3.64	0.645
Hypertension	52 (61.2%)	37 (64.9%)	0.652	33 (61.1%)	0.994	59 (70.2%)	0.215
Diabetes	24 (28.2%)	12 (21.1%)	0.335	16 (29.6%)	0.860	29 (34.5%)	0.378
MMSE	25.64 ± 2.68	25.33 ± 2.86	0.522	24.67 ± 2.64	0.038	24.39 ± 2.42	0.002
LSNS	36.11 ± 4.80	32.88 ± 4.32	0.000	33.46 ± 4.26	0.001	34.58 ± 5.23	0.050
SLE	0.74 ± 0.79	1.91 ± 1.20	0.000	1.69 ± 1.18	0.000	0.82 ± 0.79	0.511
GDS	2.36 ± 1.50	8.96 ± 1.77	0.000	9.09 ± 1.73	0.000	8.62 ± 1.96	0.000
Grade of PWMH	0.94 ± 0.92	1.14 ± 0.95	0.214	1.30 ± 1.06	0.038	1.56 ± 0.97	0.000
Grade of DWMH	0.86 ± 0.79	0.96 ± 0.82	0.441	1.06 ± 0.92	0.182	1.54 ± 0.94	0.000
Prevalence of SBI	28 (32.9%)	17 (29.8%)	0.696	25 (46.3%)	0.114	47 (56.0%)	0.003
Prevalence of microbleeds in							
Any region	20 (23.5%)	13 (22.8%)	0.920	15 (27.8%)	0.574	35 (41.7%)	0.012
Left hemisphere	10 (11.8%)	7 (12.3%)	0.926	12 (22.2%)	0.100	28 (33.3%)	0.001
Right hemisphere	14 (16.5%)	10 (17.5%)	0.721	8 (14.8%)	0.794	20 (23.8)	0.234
Infratentorial	9 (10.6%)	5 (8.8%)	0.722	7 (13.0%)	0.669	13 (15.5%)	0.345
Lobar	10 (11.8%)	8 (14.0%)	0.690	11 (20.4%)	0.167	27 (32.1%)	0.001
Deep	14 (16.5%)	11 (19.3%)	0.665	9 (16.7%)	0.976	22 (26.2%)	0.123

EOD: early-onset depression; POD: presenile-onset depression; LOD: late-onset depression; MMSE: minimental state examination; LSNS: Lubben Social Network Scale; SLE: Scale of Life Events; GDS: Geriatric Depression Scale; PWMH: periventricular white matter hyperintensities; DWMH: deep white matter hyperintensities; SBI: silent brain infarction.

**Table 2:** Logistic regressions about the determinants of LOD.

	OR (95% CI)	*P*
Model 1		
Female	1.390 (0.703–2.749)	0.344
Grade of PWMH	1.141 (0.711–1.830)	0.586
Grade of DWMH	1.866 (1.132–3.076)	0.014
Prevalence of SBI	1.980 (0.999–3.924)	0.050
Prevalence of microbleeds in any region	1.593 (0.750–3.383)	0.225
Model 2		
Prevalence of SBI	1.969 (0.986–3.935)	0.055
Prevalence of microbleeds in the left hemisphere	2.660 (1.122–6.309)	0.026
Model 3		
Prevalence of SBI	1.916 (0.962–3.818)	0.065
Prevalence of lobar microbleeds	2.502 (1.044–5.997)	0.040

Results about sex and WMH in Models 2 and 3 were similar to those in Model 1 and thus were not shown here. Abbreviations were explained below Table 1.

**Table 3:** Linear regression about the determinants of symptom severity in LOD.

	*B* ± SE	*P*
Age	−0.082 ± 0.049	0.100
Female	0.058 ± 0.413	0.888
Education year	−0.068 ± 0.057	0.238
Hypertension	0.082 ± 0.468	0.862
Diabetes	−0.166 ± 0.447	0.712
Degree of PWMH	−0.092 ± 0.289	0.752
Degree of DWMH	0.534 ± 0.262	0.045
Prevalence of SBI	0.036 ± 0.423	0.933
Grade of microbleeds	0.789 ± 0.196	0.000
MMSE	0.009 ± 0.093	0.924
LSNS	0.050 ± 0.046	0.281
SLE	0.266 ± 0.258	0.307

Abbreviations were explained below Table 1.
